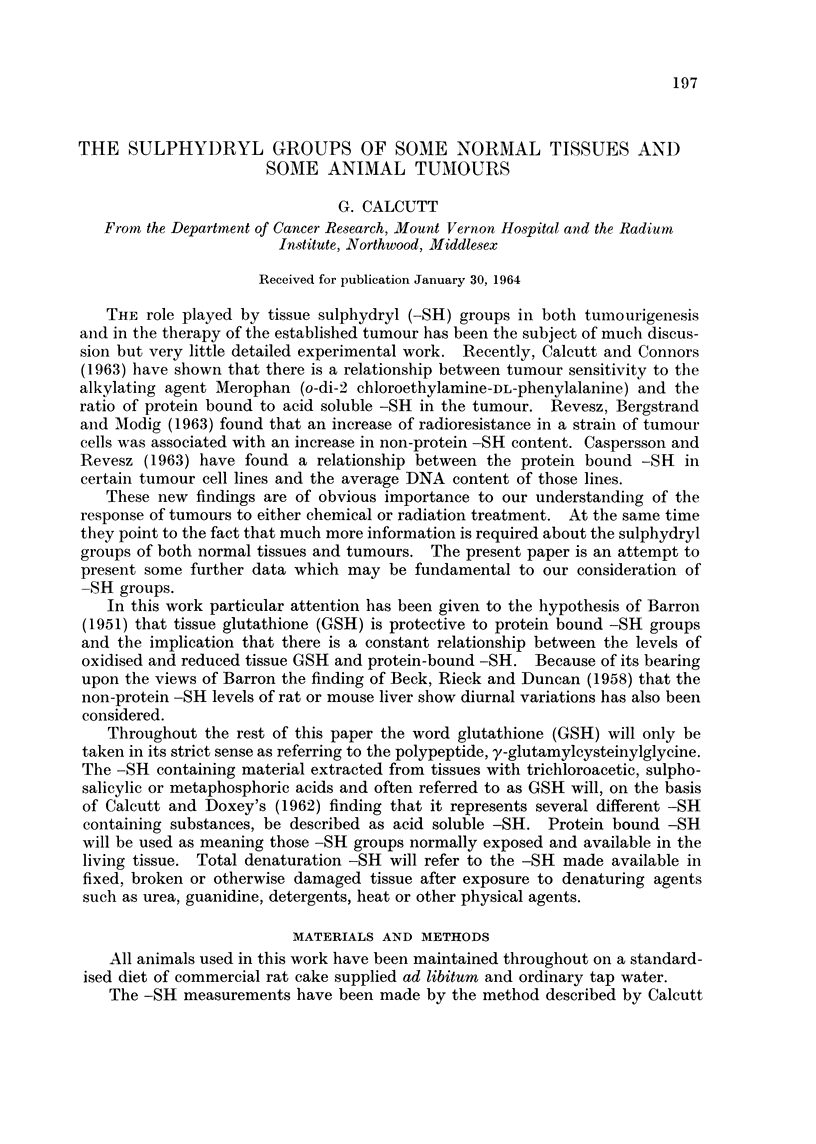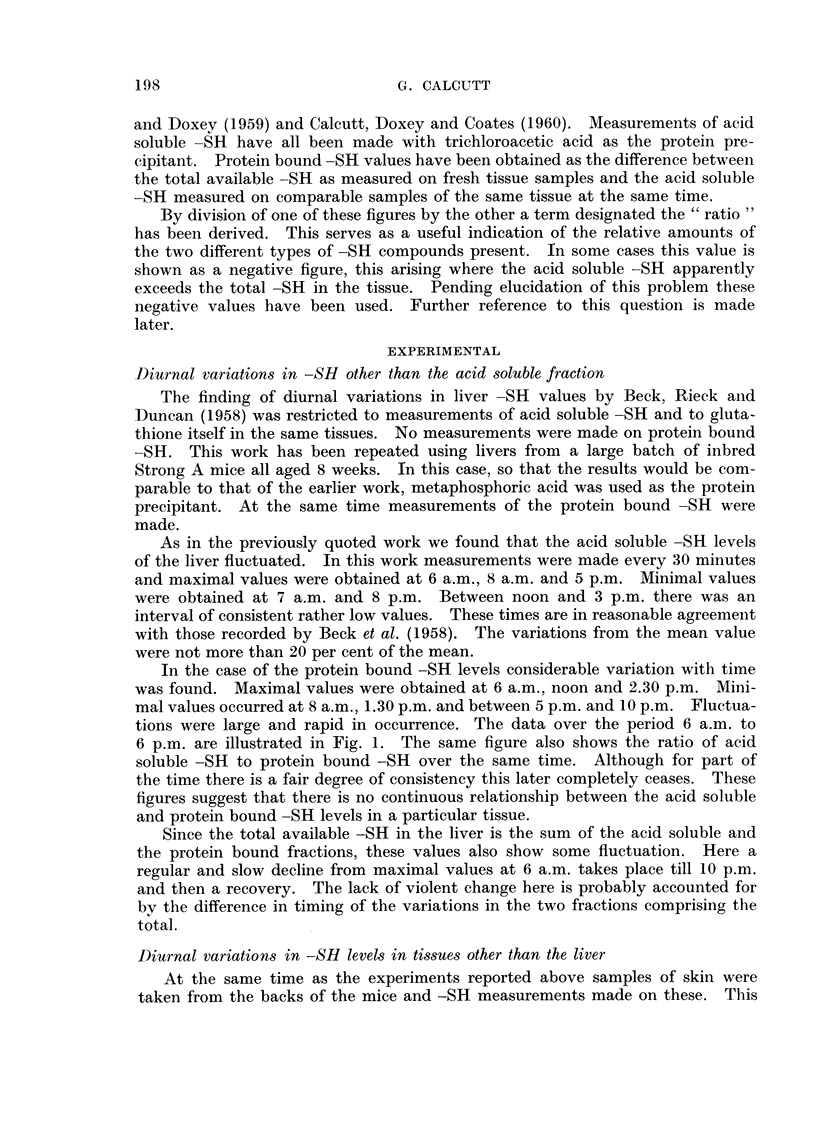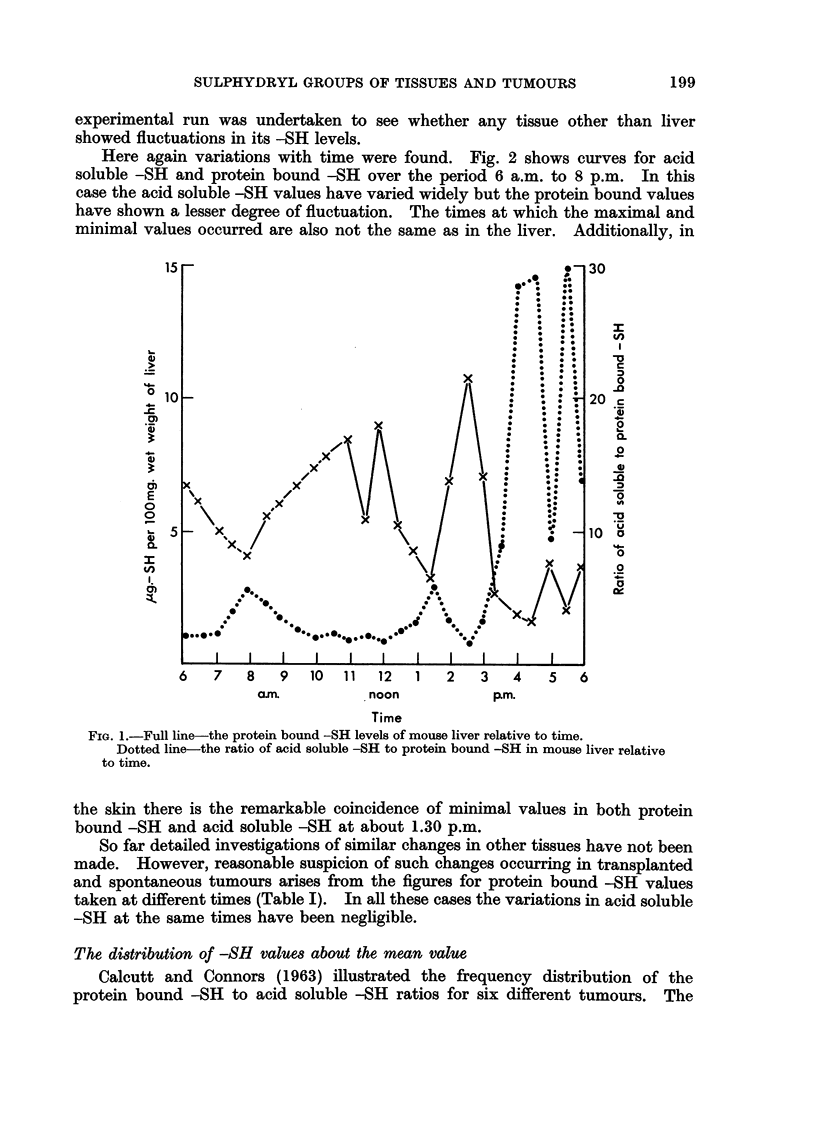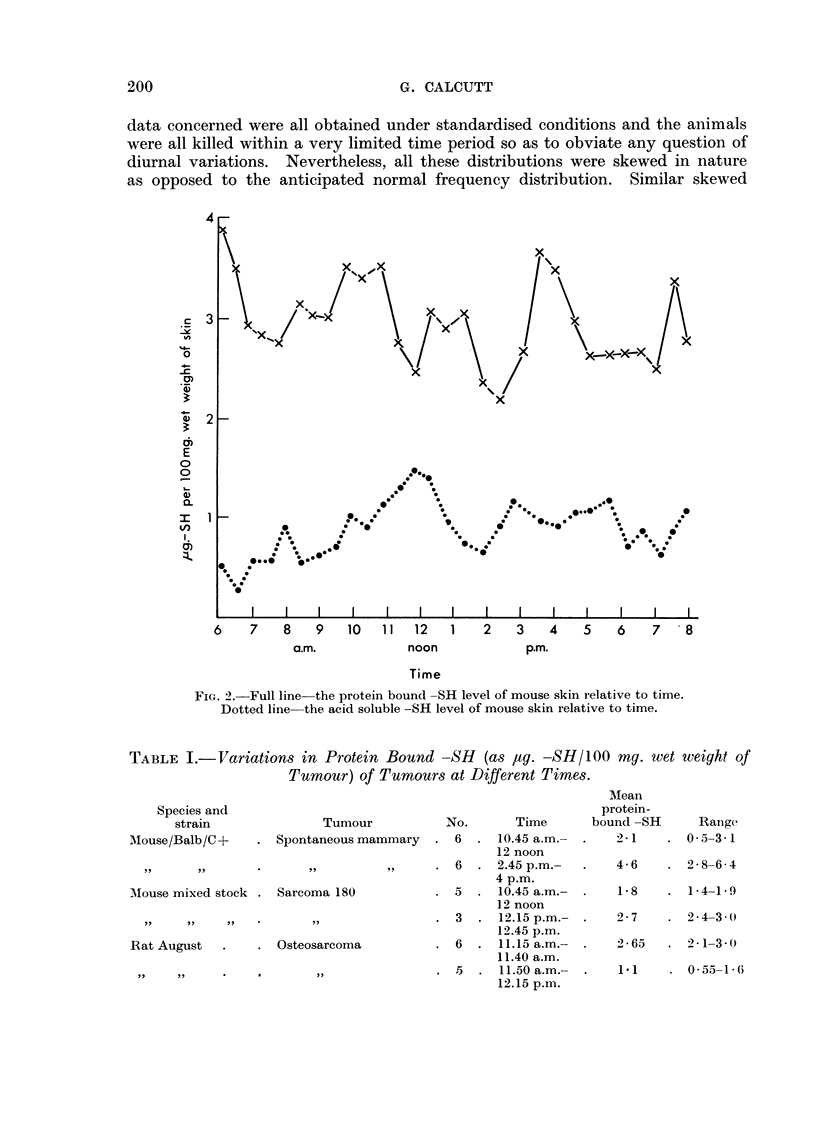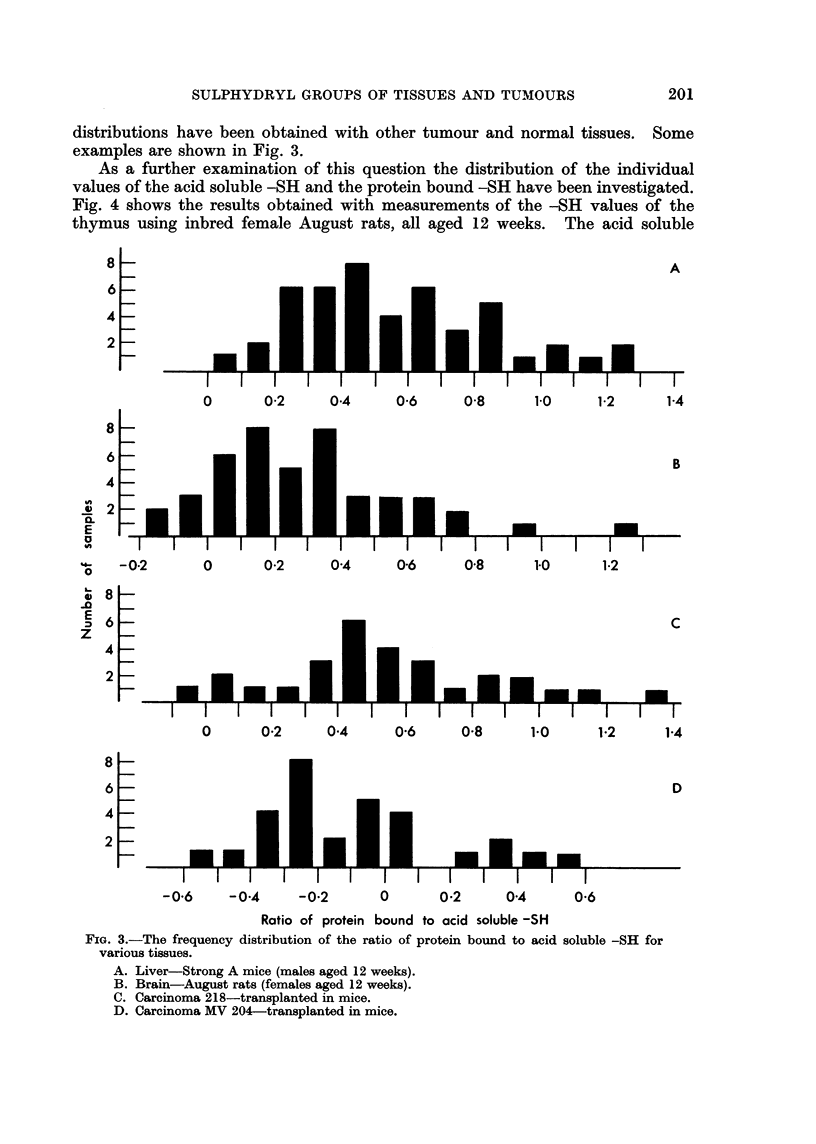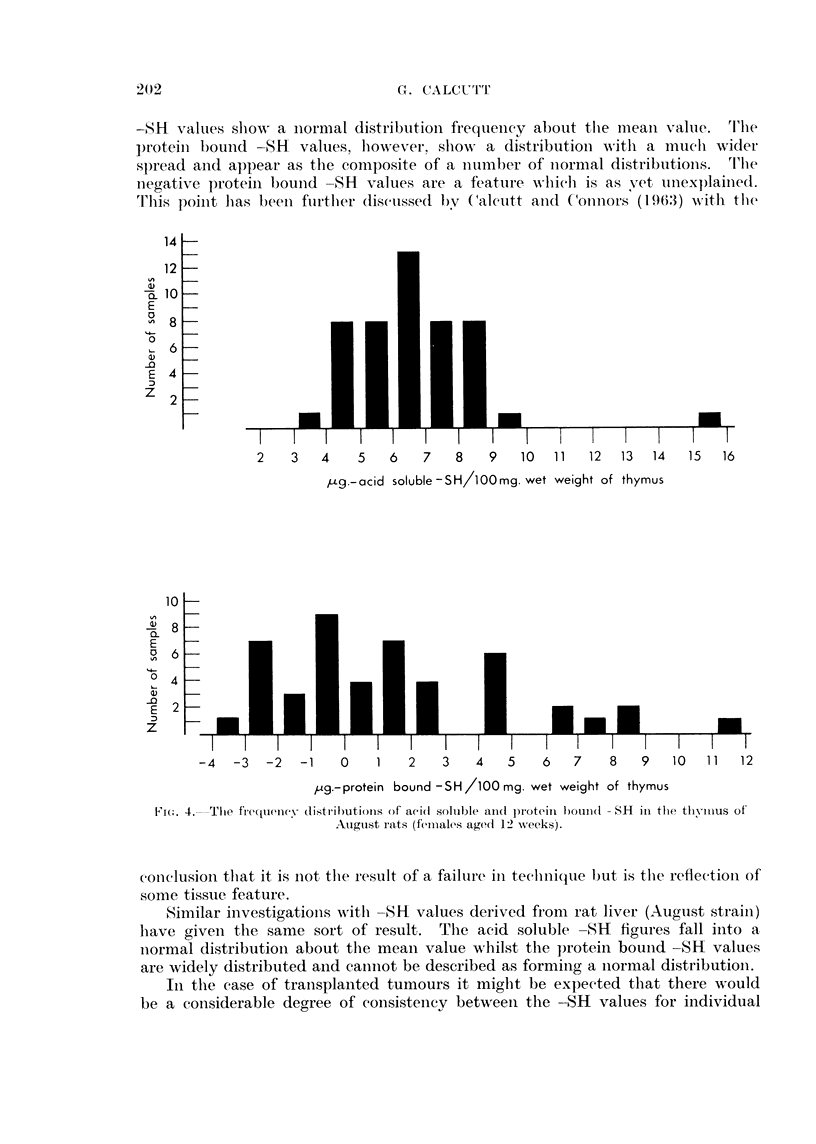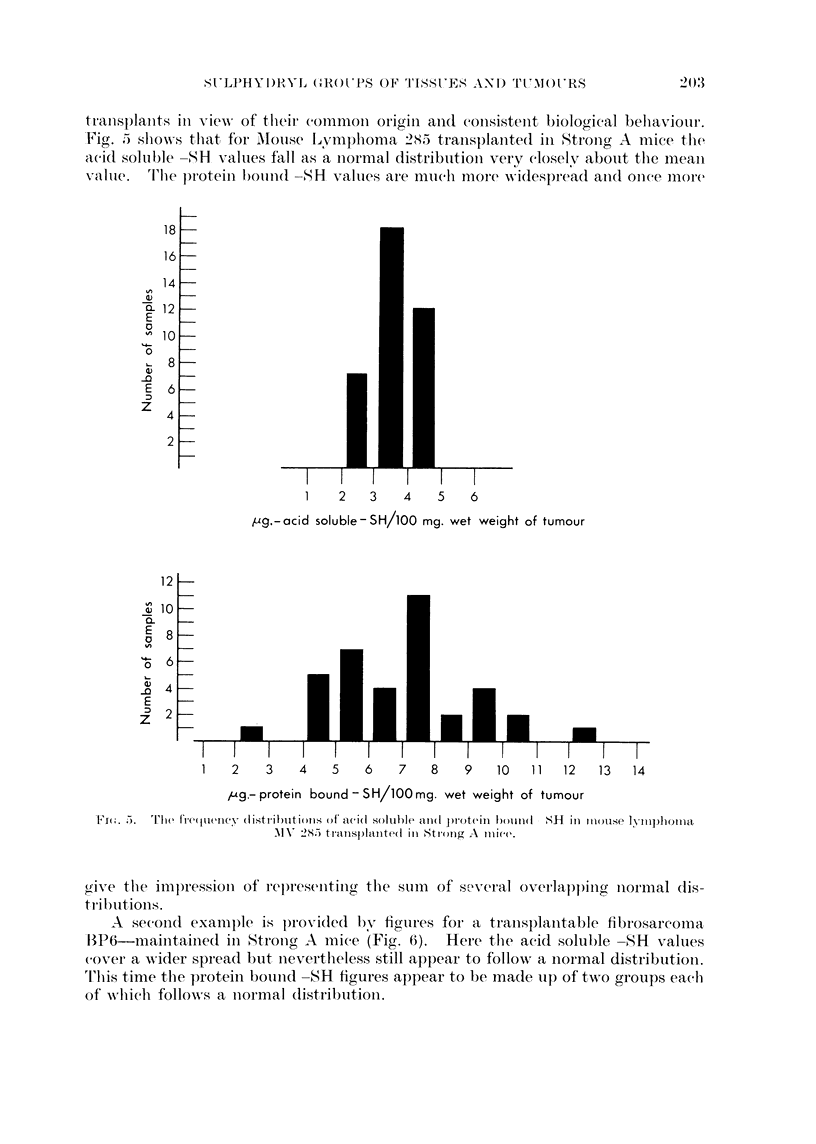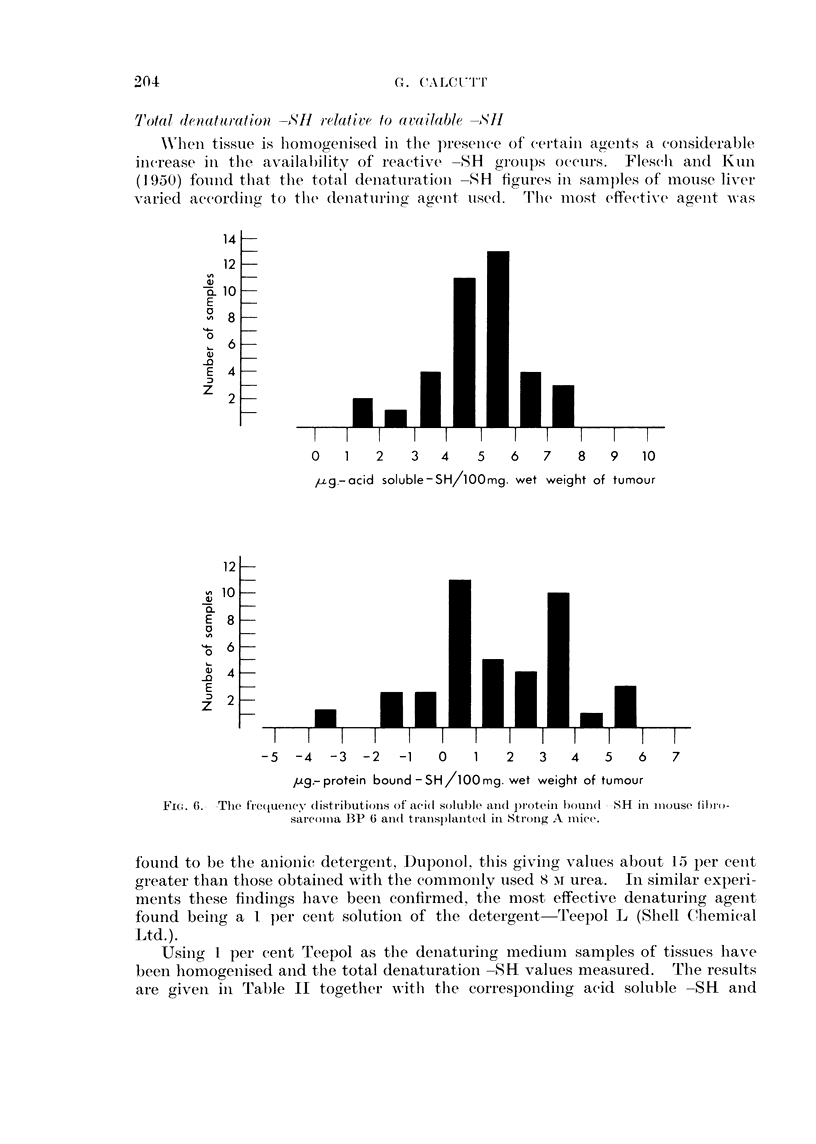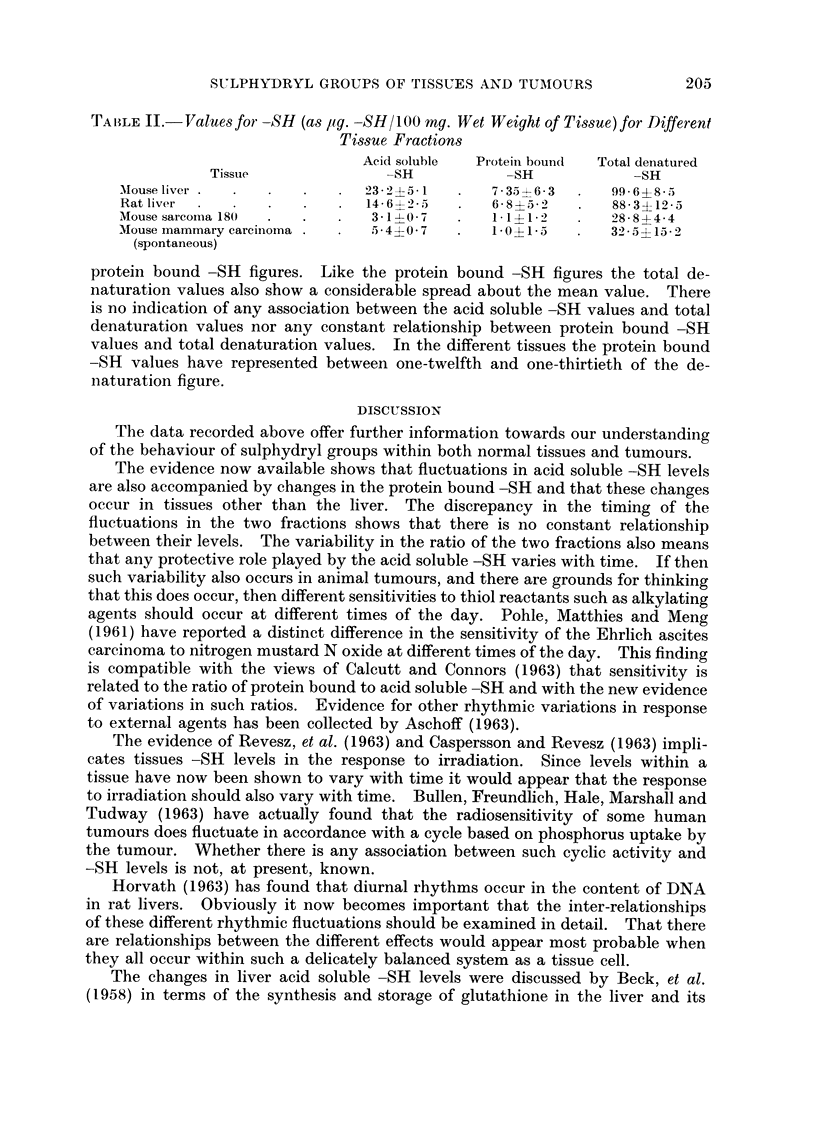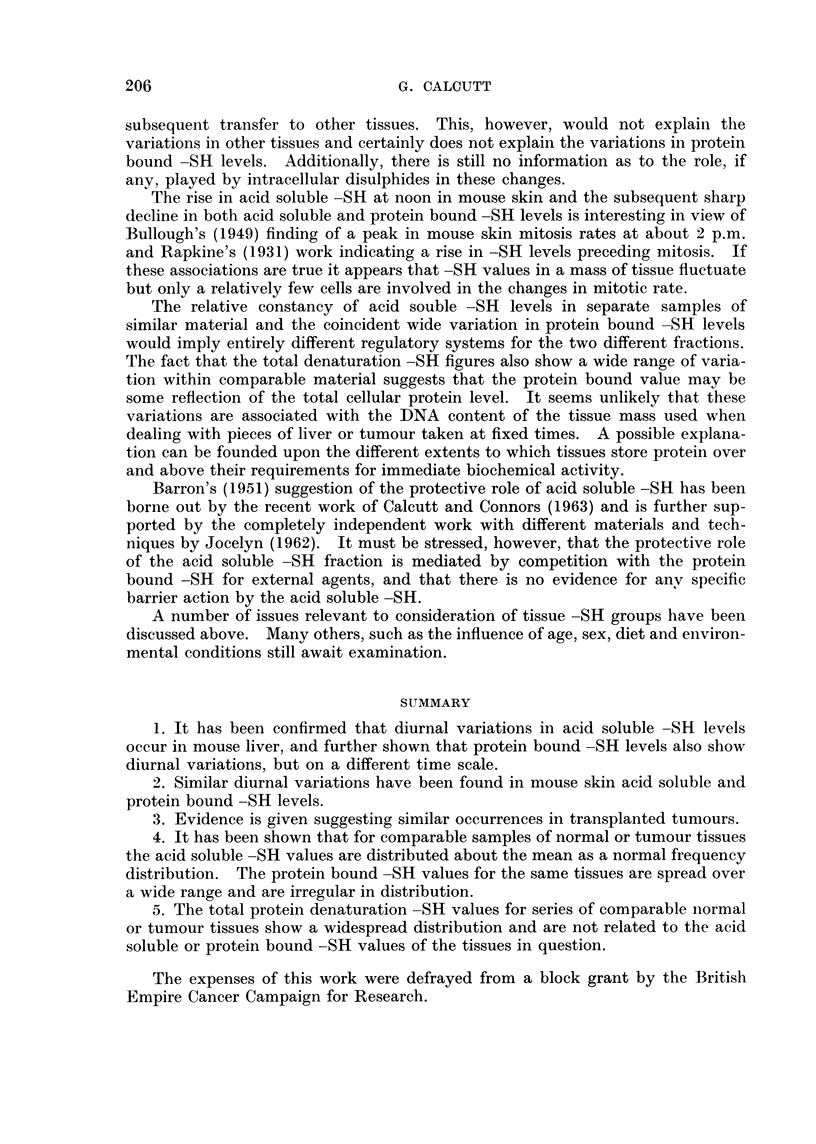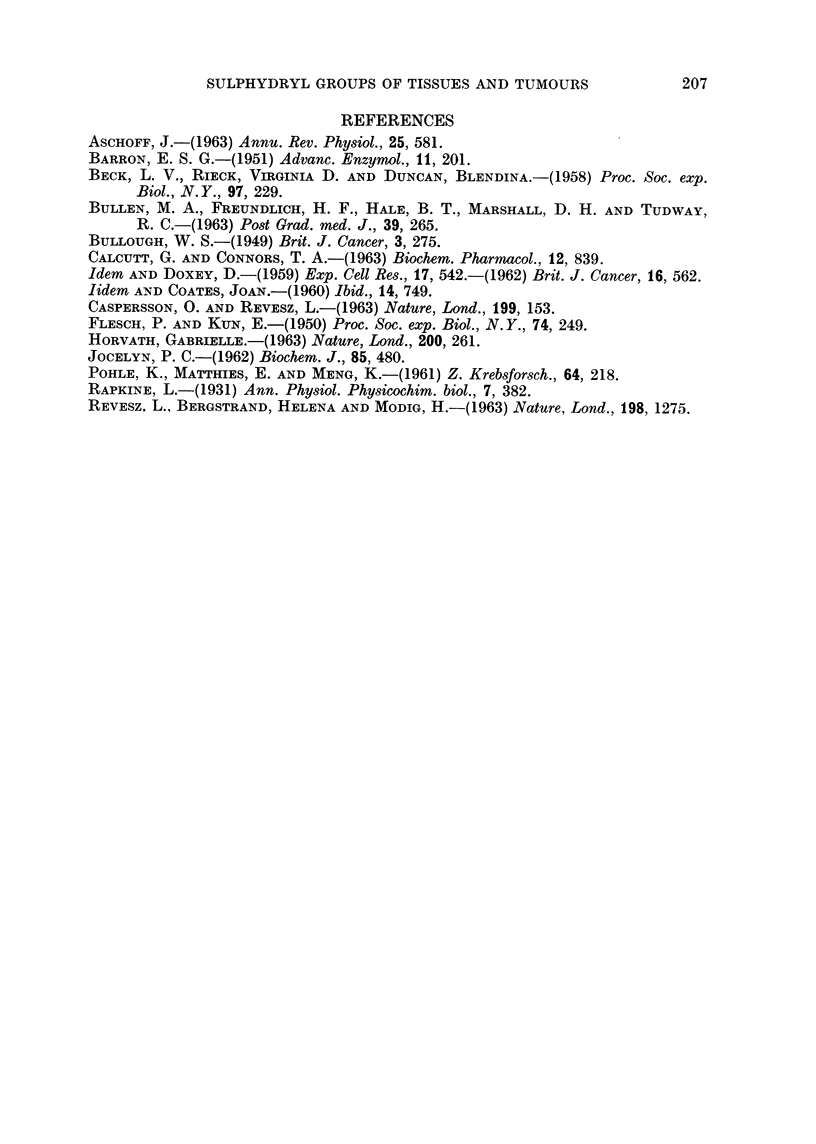# The Sulphydryl Groups of Some Normal Tissues and Some Animal Tumours

**DOI:** 10.1038/bjc.1964.22

**Published:** 1964-03

**Authors:** G. Calcutt


### THE SULPHYDRYL GROUPS OF SOME NORMAL TISSUES AND SOME ANIMAL TUMOURS

G. CALCUTT

*From the Department of Cancer Research, Mount Vernon Hospital and the Radium Institute, Northwood, Middlesex*

Received for publication January 30, 1964

THE role played by tissue sulphydryl (–SH) groups in both tumourigenesis and in the therapy of the established tumour has been the subject of much discussion but very little detailed experimental work. Recently, Calcutt and Connors (1963) have shown that there is a relationship between tumour sensitivity to the alkylating agent Merophan (*o*-di-2 chloroethylamine-DL-phenylalanine) and the ratio of protein bound to acid soluble –SH in the tumour. Revesz, Bergstrand and Modig (1963) found that an increase of radioresistance in a strain of tumour cells was associated with an increase in non-protein –SH content. Caspersson and Revesz (1963) have found a relationship between the protein bound –SH in certain tumour cell lines and the average DNA content of those lines.

These new findings are of obvious importance to our understanding of the response of tumours to either chemical or radiation treatment. At the same time they point to the fact that much more information is required about the sulphydryl groups of both normal tissues and tumours. The present paper is an attempt to present some further data which may be fundamental to our consideration of –SH groups.

In this work particular attention has been given to the hypothesis of Barron (1951) that tissue glutathione (GSH) is protective to protein bound –SH groups and the implication that there is a constant relationship between the levels of oxidised and reduced tissue GSH and protein-bound –SH. Because of its bearing upon the views of Barron the finding of Beck, Rieck and Duncan (1958) that the non-protein –SH levels of rat or mouse liver show diurnal variations has also been considered.

Throughout the rest of this paper the word glutathione (GSH) will only be taken in its strict sense as referring to the polypeptide, $\gamma$-glutamylcysteinylglycine. The –SH containing material extracted from tissues with trichloroacetic, sulphosalicylic or metaphosphoric acids and often referred to as GSH will, on the basis of Calcutt and Doxey's (1962) finding that it represents several different –SH containing substances, be described as acid soluble –SH. Protein bound –SH will be used as meaning those –SH groups normally exposed and available in the living tissue. Total denaturation –SH will refer to the –SH made available in fixed, broken or otherwise damaged tissue after exposure to denaturing agents such as urea, guanidine, detergents, heat or other physical agents.

### MATERIALS AND METHODS

All animals used in this work have been maintained throughout on a standardised diet of commercial rat cake supplied *ad libitum* and ordinary tap water.

The –SH measurements have been made by the method described by Calcutt and Doxey (1959) and Calcutt, Doxey and Coates (1960). Measurements of acid soluble –SH have all been made with trichloroacetic acid as the protein precipitant. Protein bound –SH values have been obtained as the difference between the total available –SH as measured on fresh tissue samples and the acid soluble –SH measured on comparable samples of the same tissue at the same time.

By division of one of these figures by the other a term designated the " ratio " has been derived. This serves as a useful indication of the relative amounts of the two different types of –SH compounds present. In some cases this value is shown as a negative figure, this arising where the acid soluble –SH apparently exceeds the total –SH in the tissue. Pending elucidation of this problem these negative values have been used. Further reference to this question is made later.

### EXPERIMENTAL

### *Diurnal variations in –SH other than the acid soluble fraction*

The finding of diurnal variations in liver –SH values by Beck, Rieck and Duncan (1958) was restricted to measurements of acid soluble –SH and to glutathione itself in the same tissues. No measurements were made on protein bound –SH. This work has been repeated using livers from a large batch of inbred Strong A mice all aged 8 weeks. In this case, so that the results would be comparable to that of the earlier work, metaphosphoric acid was used as the protein precipitant. At the same time measurements of the protein bound –SH were made.

As in the previously quoted work we found that the acid soluble –SH levels of the liver fluctuated. In this work measurements were made every 30 minutes and maximal values were obtained at 6 a.m., 8 a.m. and 5 p.m. Minimal values were obtained at 7 a.m. and 8 p.m. Between noon and 3 p.m. there was an interval of consistent rather low values. These times are in reasonable agreement with those recorded by Beck *et al.* (1958). The variations from the mean value were not more than 20 per cent of the mean.

In the case of the protein bound –SH levels considerable variation with time was found. Maximal values were obtained at 6 a.m., noon and 2.30 p.m. Minimal values occurred at 8 a.m., 1.30 p.m. and between 5 p.m. and 10 p.m. Fluctuations were large and rapid in occurrence. The data over the period 6 a.m. to 6 p.m. are illustrated in Fig. 1. The same figure also shows the ratio of acid soluble –SH to protein bound –SH over the same time. Although for part of the time there is a fair degree of consistency this later completely ceases. These figures suggest that there is no continuous relationship between the acid soluble and protein bound –SH levels in a particular tissue.

Since the total available –SH in the liver is the sum of the acid soluble and the protein bound fractions, these values also show some fluctuation. Here a regular and slow decline from maximal values at 6 a.m. takes place till 10 p.m. and then a recovery. The lack of violent change here is probably accounted for by the difference in timing of the variations in the two fractions comprising the total.

### *Diurnal variations in –SH levels in tissues other than the liver*

At the same time as the experiments reported above samples of skin were taken from the backs of the mice and –SH measurements made on these. This experimental run was undertaken to see whether any tissue other than liver showed fluctuations in its -SH levels.

Here again variations with time were found. Fig. 2 shows curves for acid soluble -SH and protein bound -SH over the period 6 a.m. to 8 p.m. In this case the acid soluble -SH values have varied widely but the protein bound values have shown a lesser degree of fluctuation. The times at which the maximal and minimal values occurred are also not the same as in the liver. Additionally, in

FIG. 1.—Full line—the protein bound -SH levels of mouse liver relative to time.
Dotted line—the ratio of acid soluble -SH to protein bound -SH in mouse liver relative to time.

the skin there is the remarkable coincidence of minimal values in both protein bound -SH and acid soluble -SH at about 1.30 p.m.

So far detailed investigations of similar changes in other tissues have not been made. However, reasonable suspicion of such changes occurring in transplanted and spontaneous tumours arises from the figures for protein bound -SH values taken at different times (Table I). In all these cases the variations in acid soluble -SH at the same times have been negligible.

*The distribution of -SH values about the mean value*

Calcutt and Connors (1963) illustrated the frequency distribution of the protein bound -SH to acid soluble -SH ratios for six different tumours. The data concerned were all obtained under standardised conditions and the animals were all killed within a very limited time period so as to obviate any question of diurnal variations. Nevertheless, all these distributions were skewed in nature as opposed to the anticipated normal frequency distribution. Similar skewed

FIG. 2.—Full line—the protein bound -SH level of mouse skin relative to time. Dotted line—the acid soluble -SH level of mouse skin relative to time.

TABLE I.—*Variations in Protein Bound -SH (as µg. -SH/100 mg. wet weight of Tumour) of Tumours at Different Times.*

| Species and strain | Tumour | No. | Time | Mean protein-bound -SH | Range |
|---|---|---|---|---|---|
| Mouse/Balb/C+ | Spontaneous mammary | 6 | 10.45 a.m.– 12 noon | 2·1 | 0·5–3·1 |
| ,, ,, | ,, ,, | 6 | 2.45 p.m.– 4 p.m. | 4·6 | 2·8–6·4 |
| Mouse mixed stock | Sarcoma 180 | 5 | 10.45 a.m.– 12 noon | 1·8 | 1·4–1·9 |
| ,, ,, ,, | ,, | 3 | 12.15 p.m.– 12.45 p.m. | 2·7 | 2·4–3·0 |
| Rat August | Osteosarcoma | 6 | 11.15 a.m.– 11.40 a.m. | 2·65 | 2·1–3·0 |
| ,, ,, | ,, | 5 | 11.50 a.m.– 12.15 p.m. | 1·1 | 0·55–1·6 |

distributions have been obtained with other tumour and normal tissues. Some examples are shown in Fig. 3.

As a further examination of this question the distribution of the individual values of the acid soluble –SH and the protein bound –SH have been investigated. Fig. 4 shows the results obtained with measurements of the –SH values of the thymus using inbred female August rats, all aged 12 weeks. The acid soluble

Fig. 3.—The frequency distribution of the ratio of protein bound to acid soluble –SH for various tissues.

A. Liver—Strong A mice (males aged 12 weeks).
B. Brain—August rats (females aged 12 weeks).
C. Carcinoma 218—transplanted in mice.
D. Carcinoma MV 204—transplanted in mice.

–SH values show a normal distribution frequency about the mean value. The protein bound –SH values, however, show a distribution with a much wider spread and appear as the composite of a number of normal distributions. The negative protein bound –SH values are a feature which is as yet unexplained. This point has been further discussed by Calcutt and Connors (1963) with the

FIG. 4.—The frequency distributions of acid soluble and protein bound -SH in the thymus of August rats (females aged 12 weeks).

conclusion that it is not the result of a failure in technique but is the reflection of some tissue feature.

Similar investigations with –SH values derived from rat liver (August strain) have given the same sort of result. The acid soluble –SH figures fall into a normal distribution about the mean value whilst the protein bound –SH values are widely distributed and cannot be described as forming a normal distribution.

In the case of transplanted tumours it might be expected that there would be a considerable degree of consistency between the –SH values for individual transplants in view of their common origin and consistent biological behaviour. Fig. 5 shows that for Mouse Lymphoma 285 transplanted in Strong A mice the acid soluble -SH values fall as a normal distribution very closely about the mean value. The protein bound -SH values are much more widespread and once more

Fig. 5. The frequency distributions of acid soluble and protein bound -SH in mouse lymphoma MV 285 transplanted in Strong A mice.

give the impression of representing the sum of several overlapping normal distributions.

A second example is provided by figures for a transplantable fibrosarcoma BP6—maintained in Strong A mice (Fig. 6). Here the acid soluble -SH values cover a wider spread but nevertheless still appear to follow a normal distribution. This time the protein bound -SH figures appear to be made up of two groups each of which follows a normal distribution.

*Total denaturation –SH relative to available –SH*

When tissue is homogenised in the presence of certain agents a considerable increase in the availability of reactive –SH groups occurs. Flesch and Kun (1950) found that the total denaturation –SH figures in samples of mouse liver varied according to the denaturing agent used. The most effective agent was

FIG. 6.—The frequency distributions of acid soluble and protein bound –SH in mouse fibrosarcoma BP 6 and transplanted in Strong A mice.

found to be the anionic detergent, Duponol, this giving values about 15 per cent greater than those obtained with the commonly used 8 M urea. In similar experiments these findings have been confirmed, the most effective denaturing agent found being a 1 per cent solution of the detergent—Teepol L (Shell Chemical Ltd.).

Using 1 per cent Teepol as the denaturing medium samples of tissues have been homogenised and the total denaturation –SH values measured. The results are given in Table II together with the corresponding acid soluble –SH and

TABLE II.—*Values for –SH (as μg. –SH/100 mg. Wet Weight of Tissue) for Different Tissue Fractions*

| Tissue | Acid soluble –SH | Protein bound –SH | Total denatured –SH |
|---|---|---|---|
| Mouse liver | 23·2±5·1 | 7·35±6·3 | 99·6±8·5 |
| Rat liver | 14·6±2·5 | 6·8±5·2 | 88·3±12·5 |
| Mouse sarcoma 180 | 3·1±0·7 | 1·1±1·2 | 28·8±4·4 |
| Mouse mammary carcinoma (spontaneous) | 5·4±0·7 | 1·0±1·5 | 32·5±15·2 |

protein bound –SH figures. Like the protein bound –SH figures the total denaturation values also show a considerable spread about the mean value. There is no indication of any association between the acid soluble –SH values and total denaturation values nor any constant relationship between protein bound –SH values and total denaturation values. In the different tissues the protein bound –SH values have represented between one-twelfth and one-thirtieth of the denaturation figure.

### DISCUSSION

The data recorded above offer further information towards our understanding of the behaviour of sulphydryl groups within both normal tissues and tumours.

The evidence now available shows that fluctuations in acid soluble –SH levels are also accompanied by changes in the protein bound –SH and that these changes occur in tissues other than the liver. The discrepancy in the timing of the fluctuations in the two fractions shows that there is no constant relationship between their levels. The variability in the ratio of the two fractions also means that any protective role played by the acid soluble –SH varies with time. If then such variability also occurs in animal tumours, and there are grounds for thinking that this does occur, then different sensitivities to thiol reactants such as alkylating agents should occur at different times of the day. Pohle, Matthies and Meng (1961) have reported a distinct difference in the sensitivity of the Ehrlich ascites carcinoma to nitrogen mustard N oxide at different times of the day. This finding is compatible with the views of Calcutt and Connors (1963) that sensitivity is related to the ratio of protein bound to acid soluble –SH and with the new evidence of variations in such ratios. Evidence for other rhythmic variations in response to external agents has been collected by Aschoff (1963).

The evidence of Revesz, *et al.* (1963) and Caspersson and Revesz (1963) implicates tissues –SH levels in the response to irradiation. Since levels within a tissue have now been shown to vary with time it would appear that the response to irradiation should also vary with time. Bullen, Freundlich, Hale, Marshall and Tudway (1963) have actually found that the radiosensitivity of some human tumours does fluctuate in accordance with a cycle based on phosphorus uptake by the tumour. Whether there is any association between such cyclic activity and –SH levels is not, at present, known.

Horvath (1963) has found that diurnal rhythms occur in the content of DNA in rat livers. Obviously it now becomes important that the inter-relationships of these different rhythmic fluctuations should be examined in detail. That there are relationships between the different effects would appear most probable when they all occur within such a delicately balanced system as a tissue cell.

The changes in liver acid soluble –SH levels were discussed by Beck, *et al.* (1958) in terms of the synthesis and storage of glutathione in the liver and its subsequent transfer to other tissues. This, however, would not explain the variations in other tissues and certainly does not explain the variations in protein bound –SH levels. Additionally, there is still no information as to the role, if any, played by intracellular disulphides in these changes.

The rise in acid soluble –SH at noon in mouse skin and the subsequent sharp decline in both acid soluble and protein bound –SH levels is interesting in view of Bullough's (1949) finding of a peak in mouse skin mitosis rates at about 2 p.m. and Rapkine's (1931) work indicating a rise in –SH levels preceding mitosis. If these associations are true it appears that –SH values in a mass of tissue fluctuate but only a relatively few cells are involved in the changes in mitotic rate.

The relative constancy of acid souble –SH levels in separate samples of similar material and the coincident wide variation in protein bound –SH levels would imply entirely different regulatory systems for the two different fractions. The fact that the total denaturation –SH figures also show a wide range of variation within comparable material suggests that the protein bound value may be some reflection of the total cellular protein level. It seems unlikely that these variations are associated with the DNA content of the tissue mass used when dealing with pieces of liver or tumour taken at fixed times. A possible explanation can be founded upon the different extents to which tissues store protein over and above their requirements for immediate biochemical activity.

Barron's (1951) suggestion of the protective role of acid soluble –SH has been borne out by the recent work of Calcutt and Connors (1963) and is further supported by the completely independent work with different materials and techniques by Jocelyn (1962). It must be stressed, however, that the protective role of the acid soluble –SH fraction is mediated by competition with the protein bound –SH for external agents, and that there is no evidence for any specific barrier action by the acid soluble –SH.

A number of issues relevant to consideration of tissue –SH groups have been discussed above. Many others, such as the influence of age, sex, diet and environmental conditions still await examination.

### SUMMARY

1. It has been confirmed that diurnal variations in acid soluble –SH levels occur in mouse liver, and further shown that protein bound –SH levels also show diurnal variations, but on a different time scale.

2. Similar diurnal variations have been found in mouse skin acid soluble and protein bound –SH levels.

3. Evidence is given suggesting similar occurrences in transplanted tumours.

4. It has been shown that for comparable samples of normal or tumour tissues the acid soluble –SH values are distributed about the mean as a normal frequency distribution. The protein bound –SH values for the same tissues are spread over a wide range and are irregular in distribution.

5. The total protein denaturation –SH values for series of comparable normal or tumour tissues show a widespread distribution and are not related to the acid soluble or protein bound –SH values of the tissues in question.

The expenses of this work were defrayed from a block grant by the British Empire Cancer Campaign for Research.

### REFERENCES

ASCHOFF, J.—(1963) *Annu. Rev. Physiol.*, **25**, 581.
BARRON, E. S. G.—(1951) *Advanc. Enzymol.*, **11**, 201.
BECK, L. V., RIECK, VIRGINIA D. AND DUNCAN, BLENDINA.—(1958) *Proc. Soc. exp. Biol., N.Y.*, **97**, 229.
BULLEN, M. A., FREUNDLICH, H. F., HALE, B. T., MARSHALL, D. H. AND TUDWAY, R. C.—(1963) *Post Grad. med. J.*, **39**, 265.
BULLOUGH, W. S.—(1949) *Brit. J. Cancer*, **3**, 275.
CALCUTT, G. AND CONNORS, T. A.—(1963) *Biochem. Pharmacol.*, **12**, 839.
*Idem* AND DOXEY, D.—(1959) *Exp. Cell Res.*, **17**, 542.—(1962) *Brit. J. Cancer*, **16**, 562.
*Iidem* AND COATES, JOAN.—(1960) *Ibid.*, **14**, 749.
CASPERSSON, O. AND REVESZ, L.—(1963) *Nature, Lond.*, **199**, 153.
FLESCH, P. AND KUN, E.—(1950) *Proc. Soc. exp. Biol., N.Y.*, **74**, 249.
HORVATH, GABRIELLE.—(1963) *Nature, Lond.*, **200**, 261.
JOCELYN, P. C.—(1962) *Biochem. J.*, **85**, 480.
POHLE, K., MATTHIES, E. AND MENG, K.—(1961) *Z. Krebsforsch.*, **64**, 218.
RAPKINE, L.—(1931) *Ann. Physiol. Physicochim. biol.*, **7**, 382.
REVESZ. L., BERGSTRAND, HELENA AND MODIG, H.—(1963) *Nature, Lond.*, **198**, 1275.